# Zika: The Emerging Epidemic

**DOI:** 10.3201/eid2310.171139

**Published:** 2017-10

**Authors:** Maciej F. Boni

**Affiliations:** Pennsylvania State University, University Park, Pennsylvania, USA

**Keywords:** Zika, public health response, media, viruses, United States, Brazil, vector-borne infections

In 1947, in a small forest in Uganda, near the capital, Kampala, a filterable transmissible agent was found in a sentinel rhesus macaque with a high fever. The agent was later identified as a virus and named Zika after the forest in which it was found. For the next 60 years, the virus was largely ignored by scientists because very few human cases were identified. However, in the summer of 2015, in several cities in eastern Brazil, a Zika epidemic was identified concurrently with a markedly increased number of infant microcephaly cases in maternity wards. Before long, Zika virus infection during pregnancy was identified as the cause of microcephaly, the virus was confirmed to be spreading among >40 countries in the Americas, and numerous public health agencies were faced with an emergency about how best to protect a generation of children conceived during the Zika outbreak.

The journey taken by Zika virus from Africa, across the Pacific, to the World Health Organization–declared Public Health Emergency of International Concern in February 2016 is chronicled by New York Times writer Donald G. McNeil, Jr., in Zika: The Emerging Epidemic (Figure). The book starts with the discovery of Zika virus in 1947 and traces the early research on human infections, the 2007 outbreak in Micronesia, the 2013 outbreak in French Polynesia, and the long 2015–2016 epidemic wave of Zika virus infection that occurred in the Americas. The book is organized chronologically and is current through June 2016.

**Figure:**
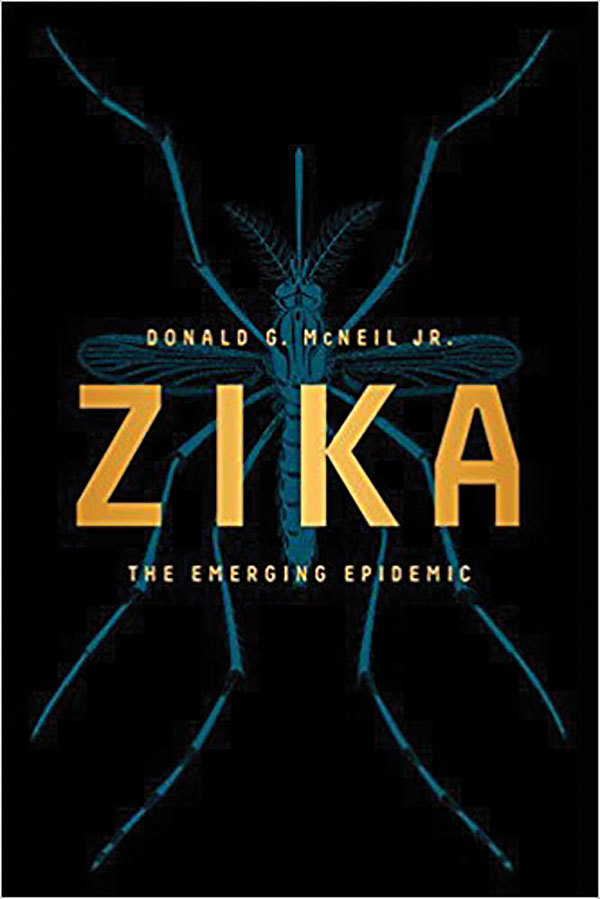
Zika: The Emerging Epidemic

McNeil’s book is thorough in its timeline and excellent at describing the political history of public health management for Zika. This history includes the blaming of an insecticide for the initial cases of Guillain-Barré syndrome in French Polynesia in 2013, the US Centers for Disease Control and Prevention’s initial handling of evidence of a sexual transmission route for Zika virus, and the controversy of whether to recommend delaying pregnancy in Zika-endemic areas where travel advisories were already in place. McNeil presents convincing arguments at the end of the book for why the recommendations to delay pregnancy should have been stronger, clearer, and more prompt. The book also catalogs a range of alternative hypotheses that emerged in the media before a scientific consensus formed that Zika did in fact cause microcephaly.

The book contains some poignant individual stories that humanize the toll of the epidemics in the Pacific and the Americas. McNeil correctly calls attention to Guillain-Barré syndrome, which typically does not generate headlines the way that a photo of a microcephalic infant does. In some instances, anecdotes about newspaper editors and public officials draw attention away from the victims of the epidemic.

The book sometimes strays into a narrative of epidemiologist or journalist as hero, a convention that should be avoided. It contains a small number of scientific inaccuracies (e.g., biological classification of *Toxoplasma*, risk for death from dengue virus), and academic readers would be wise to follow their noses to the source material, much of which McNeil lists by chapter at the end of the book. The book is very readable, as expected from a New York Times writer, and it successfully presents a current-events summary of the 2015–2016 Zika epidemic and what the world’s public health systems did and did not do to minimize the risk for those in its path.

